# Effect of Starting Powder Particle Size on the Thermoelectric Properties of Hot-Pressed Bi_0.3_Sb_1.7_Te_3_ Alloys

**DOI:** 10.3390/ma17020318

**Published:** 2024-01-08

**Authors:** Ioanna Ioannou, Panagiotis S. Ioannou, Theodora Kyratsi, John Giapintzakis

**Affiliations:** Department of Mechanical and Manufacturing Engineering, University of Cyprus, Nicosia 2901, Cyprus; gianna1992@live.com (I.I.); ioannou.s.pan@gmail.com (P.S.I.); kyratsi@ucy.ac.cy (T.K.)

**Keywords:** thermoelectric materials, mechanical alloying, nanocomposite, ball-milling, particle size, bismuth antimony telluride

## Abstract

P-type Bi_0.3_Sb_1.7_Te_3_ polycrystalline pellets were fabricated using different methods: melting and mechanical alloying, followed by hot-press sintering. The effect of starting powder particle size on the thermoelectric properties was investigated in samples prepared using powders of different particle sizes (with micro- and/or nano-scale dimensions). A peak *ZT* (350 K) of ~1.13 was recorded for hot-pressed samples prepared from mechanical alloyed powder. Moreover, hot-pressed samples prepared from ≤45 μm powder exhibited similar *ZT* (~1.1). These high *ZT* values are attributed both to the presence of high-density grain boundaries, which reduced the lattice thermal conductivity, as well as the formation of antisite defects during milling and grinding, which resulted in lower carrier concentrations and higher Seebeck coefficient values. In addition, Bi_0.3_Sb_1.7_Te_3_ bulk nanocomposites were fabricated in an attempt to further reduce the lattice thermal conductivity. Surprisingly, however, the lattice thermal conductivity showed an unexpected increasing trend in nanocomposite samples. This surprising observation can be attributed to a possible overestimation of the lattice thermal conductivity component by using the conventional Wiedemann–Franz law to estimate the electronic thermal conductivity component, which is known to occur in nanocomposite materials with significant grain boundary electrical resistance.

## 1. Introduction

In recent years, thermoelectric (TE) materials have drawn increasing attention from researchers because of their capability to reversibly convert heat into electricity. Thermoelectric technology provides an alternative solution to the energy crisis, urging the development of new and highly efficient thermoelectric materials. The conversion efficiency of TE devices depends on the materials’ dimensionless TE figure of merit: *ZT* = (*S*^2^·*σ*/*κ*)·*T* where *S*, *σ*, *κ* and *T* are the Seebeck coefficient, electrical conductivity, total thermal conductivity and absolute temperature, respectively. *ZT* quantifies the efficiency of a material in converting heat into electricity or vice versa. The Seebeck coefficient and electrical conductivity contribute positively to the thermoelectric performance, while thermal conductivity has a negative impact—lower thermal conductivity is desirable for higher efficiency. All these properties are interrelated and cannot be manipulated separately; thus, *ZT* optimization is extremely challenging [1,2,3].

During the past decade, nanostructuring and defect engineering have been widely applied to develop efficient thermoelectric materials [4,5,6]. Lattice defects, donor-like [7] effects and microstructural alterations were proved to be important tools in order to control charge carrier and phonon transport properties. The best commercial thermoelectric materials for applications near room temperature are still bismuth telluride-based alloys. High *ZT*s were achieved in Bi_x_Sb_2−x_Te_3_ (BST) nanocomposite materials consisting of both nano- and micro-sized particles, underlining the fact that the effective scattering of phonons and the moderately good power factor play an important role in achieving a good thermoelectric performance [8,9,10,11].

Xie et al. developed a melt-spinning technique to fabricate nanocomposites, in order to reduce thermal conductivity through the introduction of multi-scale microstructures and coherent grain boundaries [12]. As a result, a high *ZT* of 1.5 was achieved at 390 K. Another study by Dharmaiah et al. highlights the importance of grain-size and grain boundary scattering for effective phonon scattering [13]. Bi_0_._5_Sb_1_._5_Te_3_ powders of different sizes were prepared by gas atomization followed by a spark-plasma sintering process. The highest *ZT* value obtained was 1.23 at 350 K for the 32–75 μm powder bulk samples [13]. Additionally, Fan et al. reported an impressive *ZT* of 1.80 reached by the nanocomposite Bi_0_._4_Sb_1_._6_Te_3_ consisting of 40 wt% nano-inclusions [7]. Nanocomposites were obtained via melt spinning, and micron-size particles were obtained via solid state reaction. The thermal conductivity of these materials was low due to the effective scattering of phonons and led to a high figure of merit. However, Dharmaiah et al. [13] and Fan et al. [7] did not provide any information about the orientation of the samples and the direction of measurements, which are very important due to the anisotropic nature of BST material.

In this work, p-type Bi_0.3_Sb_1.7_Te_3_ polycrystalline pellets were fabricated using two different methods for the preparation of the starting powders: (i) melting followed by hand grinding or ball milling and (ii) mechanical alloying. In both cases, hot-press sintering was applied for the powder consolidation step. The particular composition was selected based on the results of previously published works, which revealed that Bi_0.3_Sb_1.7_Te_3_ is the most efficient member of the solid solution series Bi_2−x_Sb_x_Te_3_ [14,15,16,17]. This work aims to investigate the effect of starting powder particle size on the thermoelectric properties and the potential of their controlled selection on the development of high-performance materials.

## 2. Materials and Methods

Bi_0.3_Sb_1.7_Te_3_ samples were prepared via two different methods, (a) melting and (b) mechanical alloying. 

(a) Melting followed by hand grinding was used to obtain powders consisting of micron-sized particles. High-purity (5N) Bi, Sb and Te granules were weighed according to the stoichiometric ratio of Bi_0.3_Sb_1.7_Te_3,_ and then an excess amount of Te (4 wt%) was added to the mixture in order to compensate for its loss during heating due to its low vapor point (550 K at ${133.3*10}^{-4\;}Pa$) and, hence, ensure that the resulting material would possess the targeted composition. The metal mixture was loaded into an evacuated quartz-sealed tube and melted at temperatures over 1073 K for 10 h. At the end of the heating cycle, the melt was slowly cooled down to room temperature. The ingot (diameter = 10 mm, length ≈ 40 mm) obtained from melting was hand ground to obtain micron-sized particles, and an amount of this powder was further ball-milled under Ar atmosphere to obtain powders consisting of nano-sized crystallites. The milling was carried out in a planetary mill with an effective diameter of main disk of 121.9 mm at a speed of 300 rpm for 20 h, and the ball-to-material ratio was 10:1. Hand-ground powders were divided into four groups depending on their particles size by using sieves. An amount of powder produced by melting, and hand grinding was ball-milled in order to produce nano-powders. 

(b) Mechanical alloying was used to obtain nano-powders in order to compare the results with the melting-ball-milling method. High-purity (5N) Bi, Sb and Te elements were weighed following the nominal compositions, in a glovebox under Ar atmosphere and were loaded in a tungsten carbide vial along with 10 mm balls. The milling process was carried out in a planetary mill for 20 h at 300 rpm. 

**Figure 1:**
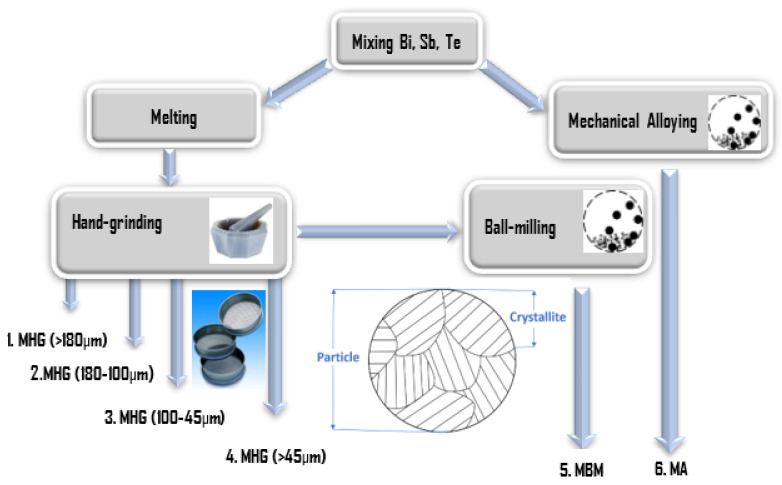
Schematic presentation of the preparation of Bi_0.3_Sb_1.7_Te_3_ powders with different particle sizes.

For brevity, samples prepared via melting and hand grinding will be noted as MHG, samples prepared via melting and ball-milling will be noted as MBM and samples prepared via mechanical alloying will be noted as MA. In summary, six types of powders with different particle sizes were prepared, as illustrated in Figure 1. 

Then, the powders were loaded in a cylindrical graphite die (inside diameter = 10 mm) and hot-pressed at 683 K for one hour under an axial pressure of 80 MPa. The phase, crystallite size and level of preferred orientation were investigated via X-ray diffraction (XRD) using a 9 kW rotating anode Rigaku SmartLab diffractometer (Rigaku, Tokyo, Japan). The temperature dependence of all thermoelectric properties (electrical conductivity, thermal conductivity and Seebeck coefficient) was measured along the same in-plane direction and reliable thermoelectric power factor and *ZT* values were calculated. 

The thermal conductivity was calculated using the relation *κ* = *D.ρ.*$C_{p}$, where *D* is the thermal diffusivity, *ρ* is the density and $C_{p}$ is the specific heat at constant pressure of the sample. The in-plane thermal diffusivity (*D*) was measured using a Netzsch Laser Flash Apparatus LFA-457 system (Netzsch, Selb, Germany) using the method proposed by Xie et al. [18]. According to this method, the samples were cut into four bars and then glued together after being rotated 90° counterclockwise. This re-configuration allowed for the measurement of *D* along the in-plane direction (perpendicular to the hot-press direction), the same direction in which the Seebeck coefficient and electrical resistivity were measured. The *ρ* of the samples was calculated from their measured dimensions and mass. The $C_{p}$ was taken from the literature [19,20]. The in-plane electrical conductivity and Seebeck coefficient were simultaneously measured by a standard four-probe method (ZEM-3, Ulvac-Riko, Yokohama, Japan). The carrier concentration (*n_H_*) and the Hall mobility (*μ*) at room temperature were measured using the Van der Pauw technique under a magnetic field of 2 T and a dc current of 20 mA in a Physical Properties Measurement System (PPMS, Quantum Design, San Diego, CA, USA).

## 3. Results

### 3.1. Bi_0.3_Sb_1.7_Te_3_ Alloys Prepared from Powders with Different Particle Sizes

#### Structural Characterization

**Figure 2:**
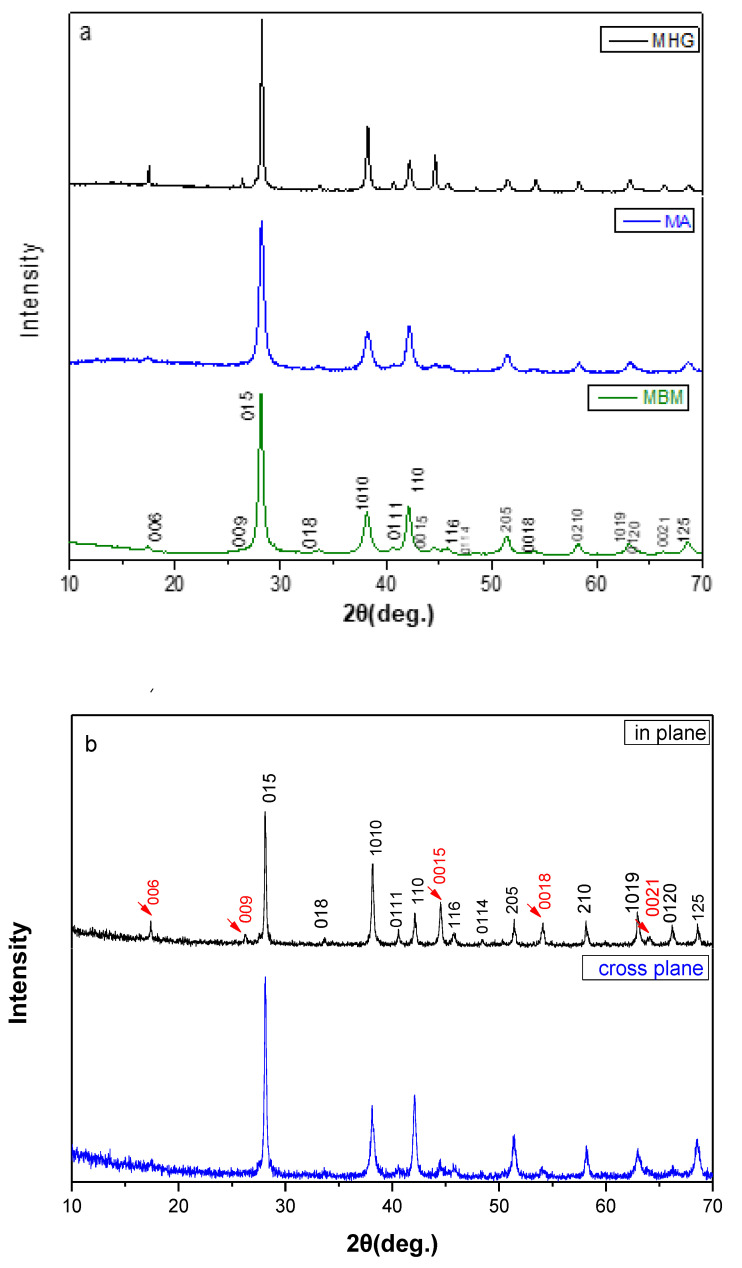

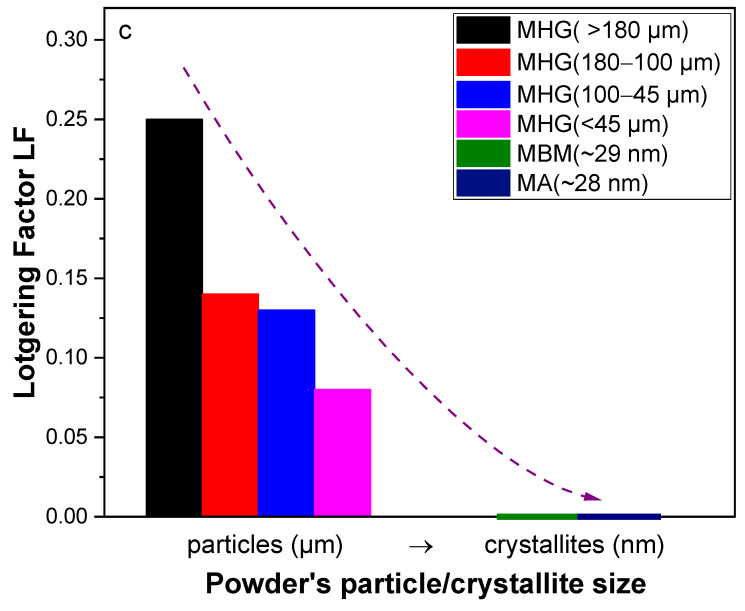
XRD diffraction patterns for (**a**) the powders produced by MGH, MA and MBM, and (**b**) the hot-pressed Bi_0.3_Sb_1.7_Te_3_ samples prepared via MGH for both in-plane and cross-plane direction. The red arrows correspond to (00*l*) peaks. (**c**) The Lotgering factor (*LF*) of the Bi_0.3_Sb_1.7_Te_3_ hot-pressed samples prepared via MGH, MBM and MA.

(A) Powders to be pressed:

Figure 2a displays XRD patterns of the powders produced by melting (MHG), mechanical alloying (MA) and ball-milling (MBM). The Miller indices of all major peaks are indicated, and all observed diffraction peaks were indexed to the rhombohedral crystal structure corresponding to Bi_0.3_Sb_1.7_Te_3_ (R3m space group). 

The Scherrer formula was used to calculate the mean crystallite size of the powders fabricated by MBM and MA:

$$\beta \left(2\Theta \right)=\frac{Κ\lambda }{Lcos\Theta }$$

where *L* is the mean grain size, *K* is a dimensionless shape factor with a value of about 0.9, *λ* is the X-ray wavelength, Θ is the Bragg angle and *β* is the line broadening at half the maximum intensity (*FWHM*) after subtracting the instrumental broadening. Line broadening refers to the apparent widening of the X-ray diffraction peaks, and it provides valuable insights into the structural characteristics of crystalline materials. This phenomenon is often a result of several factors, such as the size of the crystalline domains, defects and strains within the crystal lattice. By subtracting the instrumental broadening, the intrinsic broadening of the diffraction peaks due to these structural features was isolated.

After applying the Scherrer formula to the diffraction data, the mean crystallite size was calculated to be 28 ± 3 nm and 29 ± 4 nm for powders produced by MA and MBM, respectively.

(B) Hot-pressed pellets:

Figure 2b shows a typical XRD diffraction pattern of a Bi_0.3_Sb_1.7_Te_3_ sample in two directions, namely, the in-plane direction (the X-ray beam was incident on the surface of the sample that was perpendicular to the hot-press direction) and the cross-plane direction (the X-ray beam was incident on the surface of the sample that was parallel to the hot-press direction). All diffraction peaks are well matched to the desired Bi_0.3_Sb_1.7_Te_3_ phase. 

The existence of preferred orientation in the in-plane configuration is revealed by the (00*l*) diffraction intensities, which are higher than the ones in the cross-plane pattern. The degree of preferred orientation was evaluated by calculating the Lotgering factor (*LF*) [21]:

$$LF=\frac{P-P_{O}}{1-P_{O}},\;P=\frac{\sum I(00l)}{\sum I(hkl)},\;P_{O}=\frac{\sum I_{o}(00l)}{\sum I_{o}(hkl)},$$

where *I_o_*(*hkl*) and *I*(*hkl*) are the peak intensities of a randomly oriented sample and the measured sample, respectively.

As shown in Table 1 and Figure 2c, the *LF* increases from zero, for MA and MBM samples, which is indicative of randomly oriented samples, to 0.25 for MHG (>180 μm) samples, which clearly indicates a rather strong microstructural anisotropy. SEM analysis of fractured MA samples, as presented in our recent publication [17], confirmed the randomly oriented nature of the microstructure in these materials, further supporting the XRD results.

Moreover, samples prepared via melting and especially MHG (>180 μm) and MHG (180–100 μm) exhibit higher relative density (*ρ* ~ 94%) than other samples. Specifically, ΜΒΜ samples consisting of nano-crystallites illustrate the lowest relative density, around 89%, suggesting a higher level of porosity that is consequently expected to affect the thermoelectric properties.

### 3.2. Thermoelectric Properties

**Figure 3:**
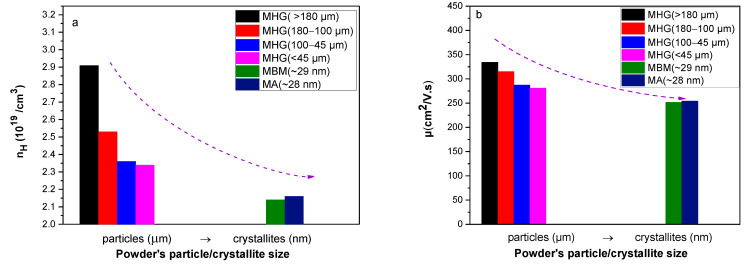
(**a**) Carrier concentration (*n_H_*) and (**b**) carrier mobility (*μ*) at room temperature as a function of powder particle crystallite size of the hot-pressed Bi_0.3_Sb_1.7_Te_3_ samples.

Figure 3 presents the hole concentration (*n_H_*) and carrier mobility (*μ*) as a function of starting powder particle size. It is evident that both *n_H_* and *μ* monotically decrease with decreasing starting particle size and reach their lowest values for samples prepared from MA and MBM powders. Τhe observed reduction in *n_H_* with decreasing particle size can be attributed to the formation of antisite defects, as supported in the literature. Ionescu et al. proposed a related model based on the idea that grinding, sintering and pressing can cause several kinds of defects and vacancies in TE materials [22]. Additionally, it has been showed that the vacancies in Te and Bi sites produce one acceptor defect and one donor defect, respectively, and since the ratio *V_Te_*/*V_Bi_* ≥ 3/2, the number of holes is smaller than that of electrons, which explains the trend of changing the conductivity from p- to n-type after mechanical treatment [22]. The formation of Te vacancies and the small difference in electronegativity of the atoms forming the compound can lead to the formation of several antisite defects that not only result in the donation of electrons to the system but also enhance the vacancy phonon scattering that reduces the lattice thermal conductivity [23]. Navratil et al. analytically described the interaction of vacancies with the antisite defects that are present in the Bi_x_Sb_2−x_Te_3_ solid solution, which can lead to a decrease in the hole carrier concentration [24]. In this work, the decrease in *μ* with decreasing starting particle size is mainly attributed to the enhanced charge carrier scattering, likely because of the gradual absence of preferred orientation and the presence of interfaces, grain boundaries and high-density lattice defects. 

**Figure 4:**
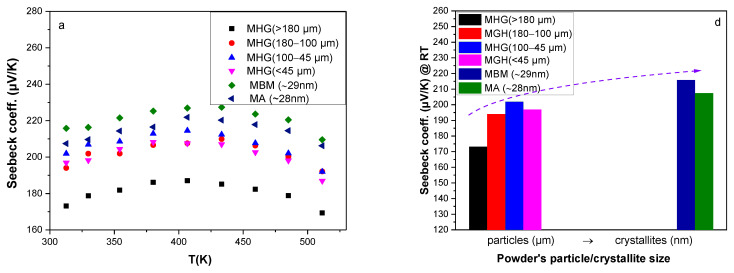

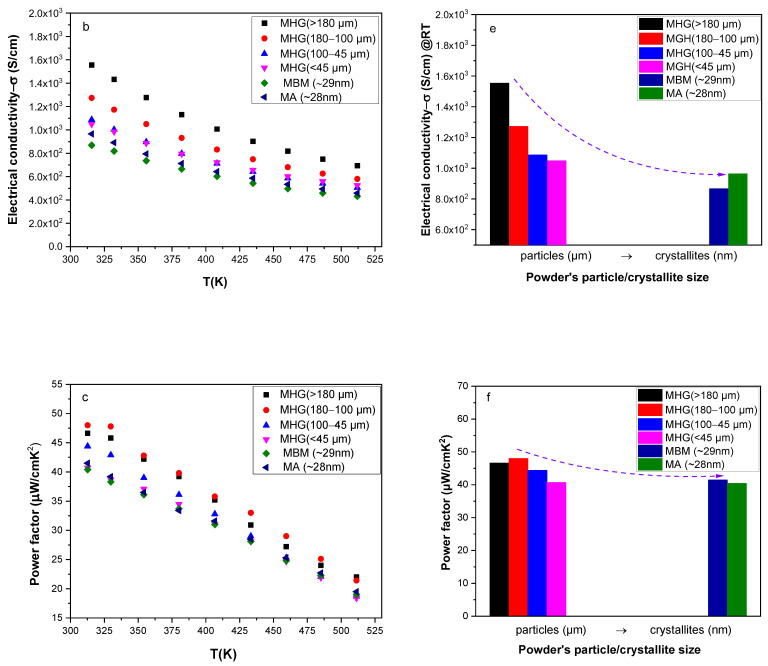
Temperature dependence of (**a**) Seebeck coefficient, (**b**) electrical conductivity and (**c**) power factor of the hot-pressed Bi_0.3_Sb_1.7_Te_3_ samples. (**d**) Seebeck coefficient, (**e**) electrical conductivity and (**f**) power factor at room temperature as a function of powder particle/crystallite size.

The temperature dependence of the Seebeck coefficient and electrical conductivity is illustrated in Figure 4a and Figure 4b, respectively. The Seebeck coefficient increases with increasing temperature, reaches a maximum value at around *T* = 400 K and then gradually decreases due to the onset of bipolar conduction (minority carriers are excited into the conduction band). The enhancement in the Seebeck coefficient for samples prepared from smaller particles is attributed to the carrier concentration reduction (as can be seen in Figure 3a). The highest Seebeck coefficient (*S* ~ 227 μV/K at 400 K) was achieved by MBM, while the sample with the largest powder particle size MHG (>180 μm) recorded the lowest value (*S* ~ 188 μV/K at 400 K). In nanostructured materials, interfacial energy barrier scattering can dominate the transport, leading to charge carrier filtering and, therefore, a higher Seebeck coefficient [25].

Electrical conductivity varies quasi-linearly with temperature (in the range of 310–520 K), indicative of a narrow-band degenerate semiconductor behavior for all studied samples. Additionally, electrical conductivity decreases for samples prepared from smaller particles. This decrease is attributed to (i) the reduction in charge carrier concentration caused by the formation of antisite defects, (ii) the reduction in hole mobility due to the presence of high-density grain boundaries, (iii) the absence of preferred orientation, especially for samples prepared via MA and MBM, and (iv) the lower density of nanostructured samples. Even though MBM and MA samples were prepared from powders consisting of similar size crystallites, they exhibit different room-temperature electrical conductivity values (see Figure 4e). Specifically, MA samples recorded slightly higher *σ* than ΜΒΜ samples. This discrepancy can be attributed to the higher relative density of MA samples, which results in higher carrier mobility, as well as tο their slightly higher hole concentration.

The temperature dependence of power factor (*PF* = *Sσ*^2^) is illustrated in Figure 4c. A maximum value of *PF* = 48.3 μWcm^−1^K^−2^ was achieved at room temperature for the MGH (180–100 μm) sample due to its high electrical conductivity. A significantly lower *PF* (~38 μWcm^−1^K^−2^) was achieved in the Bi_0.3_Sb_1.7_Te_3_ composition prepared via melting followed by ball-milling and SPS by Hu et al. [9], while Symeou et al. reported a slightly higher *PF* of 43 μW/cmK^2^ for the same composition prepared via melting and hot-press sintering [16]. Despite the high Seebeck coefficient, the *PF* of the samples prepared via mechanical alloying and ball-milling remained low (*PF* = 40 μWcm^−1^K^−2^) because of their relatively low electrical conductivity. However, these values are notably higher than those reported for other mechanically alloyed Bi_0.3_Sb_1.7_Te_3_ compositions by Jang et al. (*PF* ~ 30 μW/cmK^2^) [14] and Chen et al. (*PF* ~ 32.5 μW/cmK^2^) [15].

**Figure 5:**
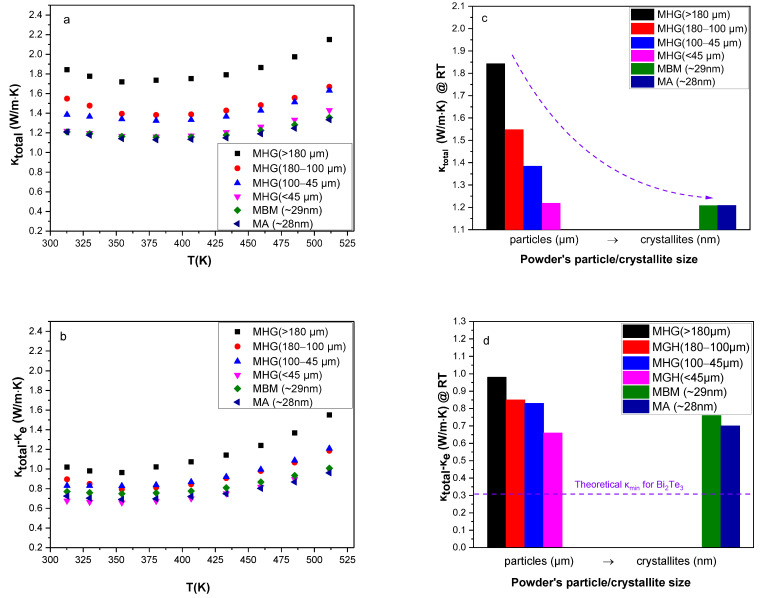
Temperature dependence of (**a**) total thermal conductivity (*κ_total_*) and (**b**) lattice and ambipolar thermal conductivity (*κ_total_* − *κ_e_*) for the hot-pressed Bi_0.3_Sb_1.7_Te_3_ samples. (**c**) *κ_total_* and (**d**) *κ_total_ − κ_e_* at room temperature as a function of particle/crystallite size.

The total thermal conductivity (*κ_total_*) as a function of temperature and starting powder particle size is presented in Figure 5a and Figure 5c, respectively. A decrease up to 38% is observed in *κ_total_* of samples prepared via MBM and MA in comparison to that of samples prepared via MHG. This is mainly attributed to the formation of several antisite defects during grinding that led to lower carrier concentration and caused a reduction of 44% in electronic thermal conductivity (*κ_e_*). The electronic contribution to the total thermal conductivity was estimated using the Wiedemann–Franz relationship (*κ_e_* = *L*·*σ*·*Τ*). The Lorenz number (*L*) was calculated from the Seebeck coefficient values by employing Fermi–Dirac statistics and taking into account acoustic phonon scattering [26,27]. In addition, the sum of the lattice thermal conductivity and the ambipolar part was estimated by subtracting the *κ_e_* from *κ_total_* and is presented as a function of temperature in Figure 5b. *κ_total_ − κ_e_* increases with temperature due to the existence of minority carriers arising with the onset of intrinsic contribution [28]. The presence of interfaces and grain boundaries as well as the abscence of preferred orientation in samples made of nano-powders (MA, MBM) enhanced the scattering of phonons and suppressed the lattice thermal conductivity by 40% [17]. As shown in Figure 5d, MHG (≤45 μm) samples exhibited the lowest lattice thermal conductivity (*κ_lattice_* ~ 0.67 W/m.K) at room temperature. The thermal conductivity value of approximately 0.67 W/m.K for the MHG (≤45 μm) sample at room temperature is notably lower, although it does not come close to the theoretical lower limit of thermal conductivity (*κ_min_* ~ 0.31 W/m.K) [10,29]. This indicates that the particles present in the initial powder, spanning a wide size range, including micron-particles under 45 μm and potentially sub-micrometer particles, play a role in reducing the phonon mean free path (MFP) and, as a result, moderate the lattice thermal conductivity. The theoretical minimum thermal conductivity *κ_min_* for Bi_2_Te_3_ was calculated using a model proposed by Cahill et al. [29]:

$$\kappa_{min}={\left(\frac{\pi }{6}\right)}^{1/3}k_{B}n^{2/3}\sum_{i}Ui{\left(\frac{T}{\Theta i}\right)}^{2}\int_{0}^{\Theta i/T}\frac{x^{3}e^{x}}{{\left(e^{x}-1\right)}^{2}}dx$$

The sum is taken over the three sound modes (two transverse and one longitudinal) with speeds of sound *Ui. Θi* is the Debye temperature for each polarization expressed in degrees *k* $(\Theta i=Ui(ћ/k_{B}){\left(6\pi^{2}n\right)}^{1/3}$), and *n* is the number density of atoms. 

According to the theory, structures composed of features of different size are expected to scatter different groups of phonon MFPs more effectively and potentially reduce the thermal conductivity down to the theoretical limits [30]. 

In numerous studies, atomistic simulations suggest that grain sizes smaller than 100 nm are necessary to reduce the lattice thermal conductivity by decreasing phonon MFPs [31,32]. This is in contrast to available experimental data, where a remarkable thermal conductivity reduction is observed, even for micro-grained Bi_2_Te_3_-based samples. In this case, micro-grains should only slightly reduce the *κ_lattice_* based on classical phonon size effects. According to a theoretical study by Wang et al. [33], the discrepancy between computed phonon MFPs and the measured *κ_lattice_* reduction in polycrystalline materials can be resolved by considering the interfacial thermal resistance (*R_K_*) [34,35] at grain boundaries, which results from the frequency-dependent phonon transmission or reflection at grain boundaries. It was found that a high *R_K_* at grain boundaries could be the main cause for the observed significant thermal conductivity reduction.

**Figure 6:**
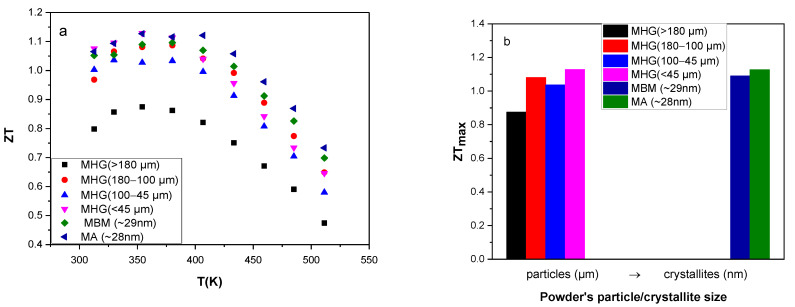
(**a**) Thermoelectric figure of merit *ZT* of the various hot-pressed Bi_0.3_Sb_1.7_Te_3_ samples as a function of temperature and (**b**) maximum *ZT* (*ZT_max_*) as a function of starting powder particle/crystallite size.

The calculated *ZTs* are presented in Figure 6. The results reveal that the best thermoelectric performance *ZT* (350 K) ~ 1.13 is obtained for two samples: the MA sample and the MHG (≤45 μm) sample. This value is almost 30% higher than that of MHG (>180 μm). In addition, the average *ZT* (*ZT_av_*) value of MA and MHG (≤45 μm) samples, over the investigated temperature region, is 1.02 and 0.96, respectively, while the *ZT_av_* value of MHG (>180 μm) is only 0.74. Although the calculated power factors of the MA and MHG (≤45 μm) samples were significantly lower than those of samples prepared from powders with larger particle sizes due to the lower electrical conductivities, their remarkably reduced thermal conductivity contributed to achieving a high *ZT*.

### 3.3. Fabrication of Nanocomposite Bulk Bi_0.3_Sb_1.7_Te_3_ Alloys

Theoretical predictions claim that the presence of randomly distributed pores and features of different size scale results in a shortening of the MFP of thermal phonons, which can cause a substantial reduction in the thermal conductivity [36,37]. Defects in the atomic scale and up to a few nanometers can act as scattering points for short-wavelength phonons. Nano-scale defects, like dislocations, alloying, nano-precipitates, large quantum dots and second-phase islands, can effectively scatter phonons of short and medium wavelengths (up to ∼100 nm). Long-wavelength phonons (up to ∼1 mm) can be scattered by micro- and mesoscale defects like grain boundaries, especially at elevated temperatures [30,38,39]. Therefore, the combination of the aforementioned defects is expected to result in a reduction in the lattice thermal conductivity down to the theoretical limits by achieving broad-wavelength scattering of phonons. 

The aim of the second part of this work was the fabrication of nano-composite materials with lower thermal conductivity values while maintaining relatively unaffected power factors compared to the constituents of the nanocomposite powders. To investigate the thermoelectric properties of nanocomposite bulk Bi_0.3_Sb_1.7_Te_3_ alloys, powders consisting of 50%MHG particles and 50%MBM crystallites were mixed in an agate mortar until homogeneous mixtures were achieved and hot-pressed under the same compaction conditions as the samples discussed previously.

#### 3.3.1. Structural Characterization

**Figure 7:**
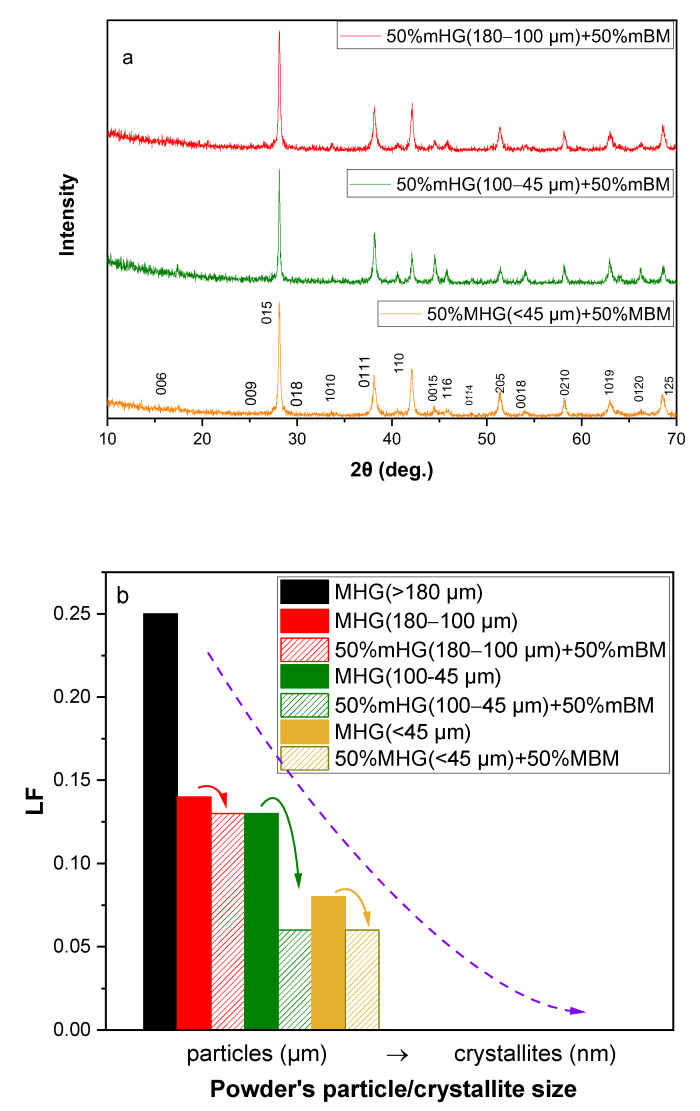
(**a**) In-plane X-ray diffraction patterns of the hot-pressed nanocomposite Bi_0.3_Sb_1.7_Te_3_ samples and (**b**) Lotgering factor (*LF*) as a function of powder particle/crystallite size.

Figure 7a shows the indexed X-ray diffraction patterns of Bi_0.3_Sb_1.7_Te_3_ composites prepared by mixing MHG and MBM powders with the Miller indices of all major peaks marked. The XRD patterns verify that all the samples exhibit the Bi_0.3_Sb_1.7_Te_3_ phase without any other secondary phase or impurity present. The level of preferred orientation (Lotgering factor; *LF*) was again calculated. As illustrated in Figure 7b and Table 2, samples prepared by mixing MHG and MBM powders exhibit lower *LF* values than samples prepared from 100% MHG powders, as expected based on the previous section of this work, which revealed that the degree of preferred orientation reduces as the particle size of MHG samples decreases. However, the reduction in *LF* is more obvious for the 50% MHG (100–45 μm) + 50% MBM sample, while the magnitude of the *LF* drop is lower for the 50% MHG (180–100 μm) + 50% MBM and 50% MHG (<45 μm) + 50% MBM samples. 

#### 3.3.2. Thermoelectric Properties

**Figure 8:**
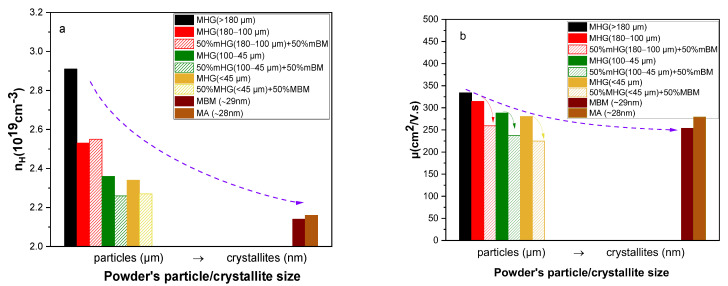
(**a**) Carrier concentration (*n_H_*) and (**b**) carrier mobility (*μ*) at room temperature as a function of powder particle/crystallite size of the hot-pressed Bi_0.3_Sb_1.7_Te_3_ samples.

Figure 8 shows the hole concentration (*n_H_*) and carrier mobility (*μ*) as a function of the initial powder particle size. As discussed in the first part of this work, MHG samples have notably higher carrier concentration than MBM and MA samples. In addition, *n_H_* monotically decreases with decreasing particle size. Thus, the combination of powders consisting of 50% nano-crystallites and 50% micro-particles is expected to decrease the carrier concentration compared to MHG samples. However, the difference in *n_H_* between the MHG (180–100 μm) sample and its nanocomposite counterpart MHG (180–100 μm) + 50% MBM is insignificant. On the other hand, 50% MHG (100–45 μm) + 50% MBM and 50% MHG (<45 μm) + 50% MBM present slightly lower *n_H_* than the anologous MHG materials, as expected. Materials prepared from mixing MBM and MHG powders revealed lower hole mobility. Specifically, MHG (180–100 μm), MHG (100–45 μm) and MHG (<45 μm) illustrate 18–20% higher *μ* in comparison to their nanocomposite counterparts. The reduction in *μ* is attributed to the enhanced hole scattering in materials originated from nm-crystallites, mainly resulting from the lower degree of preferred orientation, the higher density of interfaces and grain boundaries.

**Figure 9:**
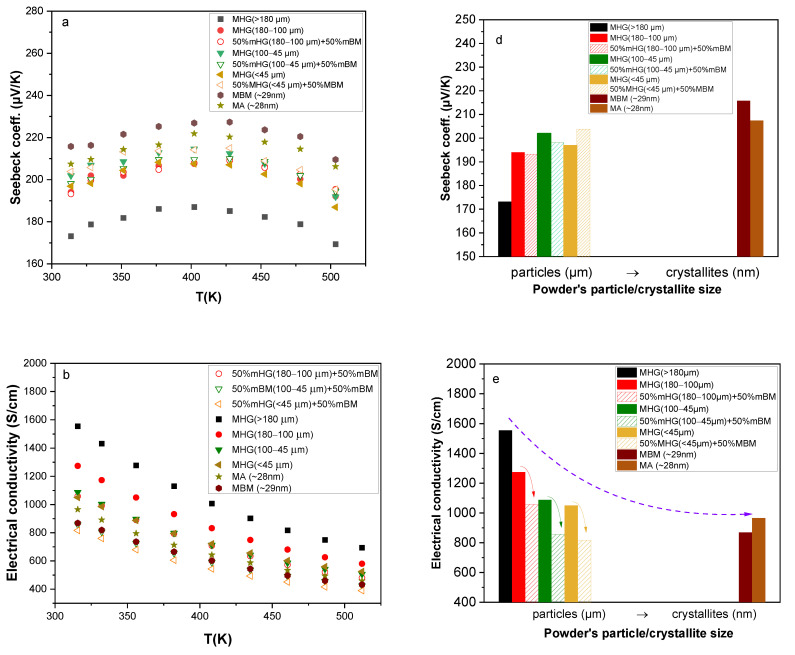

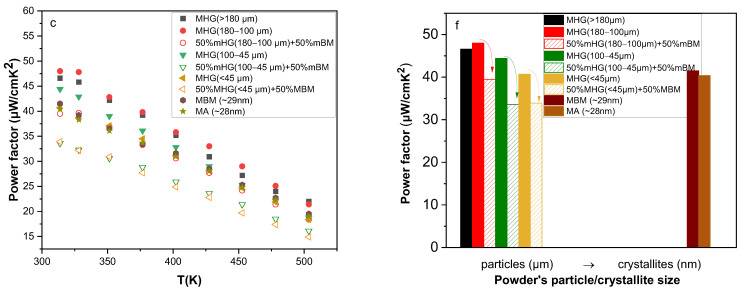
Temperature dependence of (**a**) Seebeck coefficient, (**b**) electrical conductivity and (**c**) power factor of the hot-pressed Bi_0.3_Sb_1.7_Te_3_ samples. (**d**) Seebeck coefficient, (**e**) electrical conductivity and (**f**) power factor values at room temperature as a function of powder particle/crystallite size.

The temperature dependence of the Seebeck coefficient, electrical conductivity and power factor is illustrated in Figure 9a–c, while the Seebeck coefficient, electrical conductivity and power factor values at room temperature are presented in Figure 9d–f as a function of powder particle/crystallite size. Despite the slightly lower *n_H_* values, the *S* of samples prepared from powder mixtures is about the same as that of the analogous MHG materials. Samples prepared via MBM and MA still have the highest S values, while the samples prepared from the powder with the largest particle size have the lowest *S*. The electrical conductivity (σ) of nanocomposites is significantly lower than that of the analogous MHG samples. Specifically, σ at room temperature is reduced by 17%, 21% and 22% for 50% MHG (180–100 μm) + 50% MBM, 50% MHG (100–45 μm) + 50% MBM and 50% MHG (<45 μm) + 50% MBM, respectively, in comparison to the analogous MHG samples reaching almost similar σ values as the samples prepared exclusively from nano-powders (MBM and MA). As mentioned before, *S* and *n_H_* do not significantly change after mixing MHG and MBM powders, leading us to the conclusion that the electrical conductivity reduction is a result of the hole mobility alteration. The mobility of carriers is notably reduced in samples prepared from both MHG and MBM, due to the increased scattering at the grain boundaries and the lower degree of preferred orientation, which consequently results in a lower electrical conductivity. Apart from this, in the case of 50% MHG (<45 μm) + 50% MBM, its lower density (88%) than that of its MHG counterpart could also influence the σ reduction. The calculated power factors (*PFs*) are presented in Figure 9c,f. The *PF* of 50% MHG + 50% MBM samples is severely reduced in comparison to that of their 100% MHG counterparts, reaching even lower values than MBM and MA samples. The intense reduction in *PF* is mainly attributed to the decrease in electrical conductivity.

**Figure 10:**
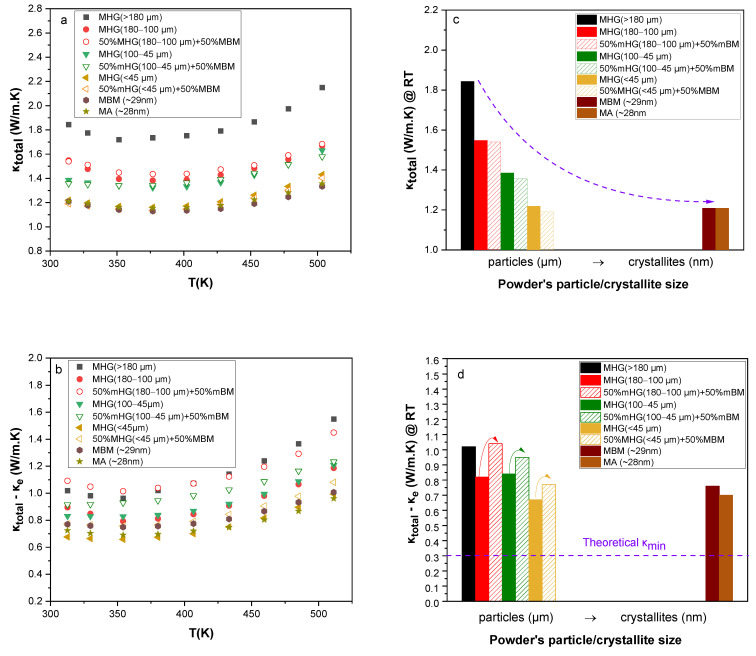
Temperature dependence of (**a**) total thermal conductivity (*κ_total_*) and (**b**) lattice and ambipolar thermal conductivity (*κ_total_* − *κ_e_*) for the hot-pressed Bi_0.3_Sb_1.7_Te_3_ samples. (**c**) *κ_total_* and (**d**) *κ_total_* − *κ_e_* at room temperature as a function of particle/crystallite size.

The total thermal conductivity (*κ_total_*) and the sum of the lattice thermal conductivity and the ambipolar part (*κ_total_* − *κ_e_*) are presented in Figure 10. Even though the electronic thermal conductivity is remarkably reduced for the 50% MHG + 50% MBM samples because of the decrease in electrical conductivity, the total thermal conductivity remains approximately the same due to the increase in lattice thermal conductivity. Normally, the scattering rates of electrons and phonons are expected to both be higher for materials with a high density of grain boundaries. Thus, the expected trend is that mobility as well as lattice thermal conductivity will drop as the grain size is reduced. In this case, *κ_lattice_* shows an unexpected increasing trend with decreasing average particle size in samples prepared by mixing MHG and MBM powders, which appears to be contradictory to the standard grain-boundary scattering theory for phonons. This unphysical inverse correlation between *κ_lattice_* and *μ* has also been observed in other thermoelectric systems, such as Mg_3_Sb_2_ [40], SnSe [41], (Hf,Zr)CoSb [42], etc. Another example is a study that investigated the effect of ball-milling duration on the grain size and thermoelectric properties of n-type Bi_2_(Te,Se)_3_ [43]. In this study, the lattice thermal conductivity of the largest-grained sample was found to be lower than that of the samples that were ball-milled for longer times and had smaller grain sizes. This observation led the investigators to conclude that this effect is probably due to grain-boundary electrical resistance [43].

The use of the conventional Wiedemann–Franz law assumes homogeneous materials, where the scattering probability is uniform everywhere, and no net exchange of energy between electrons and phonons is possible at grain boundaries or anywhere else. According to J. Kuo et al. [44], this assumption often leads to an overestimation of the phonon or lattice contribution to the thermal conductivity, especially for inhomogeneous materials, in which the length scale of the heterogeneity is larger than the MFP or the coherence length. In materials with significant grain-boundary electrical resistance, the estimated electronic contribution to the total thermal conductivity is low because the measured electrical conductivity is low. However, within the grain, electrons may still be transporting more heat than the total conductivity suggests, leading to an overestimation of *κ_lattice_* [44].

Based on the previously discussed possible overestimation of lattice thermal conductivity, we can conclude that the apparent increase in lattice thermal conductivity with mixing micron- and nano-powders is not a result of a true increase in the phonon contribution to thermal conductivity but, instead, an aftereffect of assuming homogeneous electron and phonon transport in nanocomposite materials. J. Kuo et al. [44] suggested that treating grain-boundary regions as a second phase with their own unique thermoelectric properties is essential to obtain the correct *κ_lattice_* of the material. This approximation suggests that the lattice thermal conductivity is dominated by the bulk media of the grain, which can be more precisely calculated via *κ_lattice_* = *κ* − *Lσ_G_T*, where *σ_G_* is the conductivity of the bulk grain rather than the total conductivity, as used in the conventional model.

**Figure 11:**
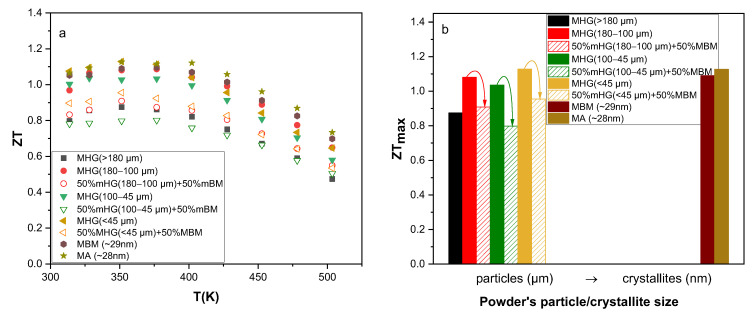
(**a**) Thermoelectric figure of merit *ZT* of the hot-pressed Bi_0.3_Sb_1.7_Te_3_ samples as a function of temperature and (**b**) maximum *ZT* (*ZT_ma_*_x_) as a function of powder particle/crystallite size.

The calculated *ZTs* are presented in Figure 11 as a function of temperature. The *ZT* of samples fabricated by mixing 50% MHG and 50% MBM powders is lower than their 100% MHG counterpart, mainly due to the significant decrease in electrical conductivity and power factor. Specifically, 50% MHG (180–100 μm) + 50% MBM, 50% MHG (100–45 μm) + 50% MBM and 50% MHG (<45 μm) + 50% MBM samples exhibit 17%, 22% and 15% lower *ZT_max_* than the analogous 100% MHG samples.

## 4. Conclusions

P-type Bi_0.3_Sb_1.7_Te_3_ bulk materials were prepared using different methods: melting and mechanical alloying. The temperature dependence of all thermoelectric properties was measured along the same in-plane direction and reliable thermoelectric power factor, and dimensionless figure-of-merit values were calculated. The experimental results indicate that materials prepared using nano-powders and powders consisting of small micron-sized particles (<45 μm) can potentially lead to significantly reduced lattice thermal conductivity, while the formation of antisite defects, caused by hand grinding and ball-milling, resulted in lower carrier concentrations and, therefore, higher Seebeck coefficient values. As a result, a *ZT* (350 K) ~ 1.13 was recorded for MA and MHG (<45 μm) samples. In the second part of this work, nanocomposite bulk Bi_0.3_Sb_1.7_Te_3_ alloys were fabricated from powders consisting of 50% MHG particles and 50% MBM crystallites in an attempt to further reduce the lattice thermal conductivity without significantly affecting the power factor. However, the notable decrease in electrical conductivity due to lower hole mobility resulted in lower *PF*s. Surprisingly, *κ_lattice_* showed an unexpected increasing trend in samples prepared by mixing MHG and MBM powders, which was contradictory to the standard grain-boundary scattering theory for phonons. This discrepancy is attributed to a possible overestimation of *κ_lattice_* by using the conventional Wiedemann–Franz law to calculate the electronic thermal conductivity in nonhomogeneous samples, which has been reported previously in nano-composite materials with significant grain-boundary electrical resistance. In summary, this work provides valuable insights by investigating the impact of fabrication methods on thermoelectric performance and addressing challenges in estimating thermal conductivity in nonhomogeneous samples. The findings offer practical implications for optimizing energy conversion devices, tailoring material properties and guiding nanocomposite approaches, while also prompting a reevaluation of theoretical frameworks for accurate thermal conductivity estimation.

## Figures and Tables

**Table 1:** Lotgering factor (*LF*), geometrical density and relative density of the hot-pressed Bi_0.3_Sb_1.7_Te_3_ samples.

Powder Type	Sample	Lotgering Factor (*LF*)	Pellet Density *ρ* (g/cm^3^)	Relative Density (%)
μm-particles	MHG (>180 μm)	0.25	6.42	94
	MHG (180–100 μm)	0.14	6.44	94
	MHG (100–45 μm)	0.13	6.11	90
	MHG (<45 μm)	0.08	6.12	90
nm-crystallites (see Figure 1)	MBM (~29 nm)	0	6.07	89
	MA (~28 nm)	0	6.29	92

**Table 2:** Lotgering factor (*LF*), geometrical density and relative density of the hot-pressed Bi_0.3_Sb_1.7_Te_3_ samples prepared using MHG, MBM, MA powders and mixtures of MHG and MBM powders.

Powder Type	Sample	Lotgering Factor (*LF*)	Pellet Density *ρ* (g/cm^3^)	Relative Density (%)
μm-particles	MHG (>180 μm)	0.25	6.42	94
	MHG (180–100 μm)	0.14	6.44	94
	MHG (100–45 μm)	0.13	6.11	90
	MHG (<45 μm)	0.08	6.12	90
both μm-particles and nm-crystallites	50%MHG (180–100 μm) + 50%MBM	0.13	6.48	95
	50%MHG (100–45 μm) + 50%MBM	0.06	6.21	91
	50%MHG (<45 μm) + 50%MBM	0.06	6.00	88
nm-crystallites (see Figure 1)	MBM (~29 nm)	0.00	6.07	89
	MA (~28 nm)	0.00	6.29	92

## Data Availability

Data available on request from the authors.

## References

[1] Rowe D.M. (1995). CRC Handbook of Thermoelectrics.

[2] Zhao L.D., Dravid V.P., Kanatzidis M.G. (2014). The panoscopic approach to high performance thermoelectrics. Energy Environ. Sci..

[3] Kanatzidis M.G. (2010). Nanostructured thermoelectrics: The new paradigm?. Chem. Mater..

[4] Liu Y., Zhou M., He J. (2016). Towards higher thermoelectric performance of Bi2Te3 via defect engineering. Scr. Mater..

[5] Liu W., Yan X., Chen G., Ren Z. (2012). Recent advances in thermoelectric nanocomposites. Nano Energy.

[6] Wu Y., Finefrock S.W., Yang H. (2012). Nanostructured thermoelectric: Opportunities and challenges. Nano Energy.

[7] Fan S., Zhao J., Guo J., Yan Q., Ma J., Hng H.H. (2010). P-type Bi_0.4_Sb_1.6_Te_3_ nanocomposites with enhanced figure of merit. Appl. Phys. Lett..

[8] Xie W., Tang X., Yan Y., Zhang Q., Tritt T.M. (2009). Unique nanostructures and enhanced thermoelectric performance of melt-spun BiSbTe alloys. Appl. Phys. Lett..

[9] Hu L.P., Zhu T.J., Wang Y.G., Xie H.H., Xu Z.J., Zhao X.B. (2014). Shifting up the optimum figure of merit of p-type bismuth telluride-based thermoelectric materials for power generation by suppressing intrinsic conduction. NPG Asia Mater..

[10] Chiritescu C., Mortensen C., Cahill D.G., Johnson D., Zschack P. (2009). Lower limit to the lattice thermal conductivity of nanostructured Bi_2_Te_3_-based materials. J. Appl. Phys..

[11] Poudel B., Hao Q., Ma Y., Lan Y., Minnich A., Yu B., Yan X., Wang D., Muto A., Vashaee D. (2008). High-thermoelectric performance of nanostructured bismuth antimony telluride bulk alloys. Science.

[12] Xie W., He J., Kang H.J., Tang X., Zhu S., Laver M., Wang S., Copley J.R.D., Brown C.M., Zhang Q. (2010). Identifying the specific nanostructures responsible for the high thermoelectric performance of (Bi,Sb)_2_Te_3_ nanocomposites. Nano Lett..

[13] Dharmaiah P., Kim H.S., Lee C.H., Hong S.J. (2016). Influence of powder size on thermoelectric properties of p-type 25%Bi_2_Te_3_–75%Sb_2_Te_3_ alloys fabricated using gas-atomization and spark-plasma sintering. J. Alloys Compd..

[14] Jang K.W., Kim H.J., Jung W.J., Kim I.H. (2018). Charge transport and thermoelectric properties of P-type Bi_2_-xSbxTe_3_ prepared by mechanical alloying and hot pressing. Korean J. Met. Mater..

[15] Chen C., Liu D.-W., Zhang B.-P., Li J.-F. (2010). Enhanced Thermoelectric Properties Obtained by Compositional Optimization in p-Type Bi_x_Sb_2−x_Te_3_ Fabricated by Mechanical Alloying and Spark Plasma Sintering. J. Electron. Mater..

[16] Symeou E., Nicolaou C., Delimitis A., Androulakis J., Kyratsi T., Giapintzakis J. (2019). High thermoelectric performance of Bi_2−x_Sb_x_Te_3_ bulk alloys prepared from non-nanostructured starting powders. J. Solid State Chem..

[17] Ioannou I., Ioannou P.S., Kyratsi T., Giapintzakis J. (2023). Low-cost preparation of highly-efficient thermoelectric Bi_x_Sb_2−x_Te_3_ nanostructured powders via mechanical alloying. J. Solid State Chem..

[18] Xie W., He J., Zhu S., Holgate T., Wang S., Tang X., Zhang Q., Tritt T.M. (2011). Investigation of the sintering pressure and thermal conductivity anisotropy of melt-spun spark-plasma-sintered (Bi,Sb)_2_Te_3_ thermoelectric materials. J. Mater. Res..

[19] Shtern Y.I., Malkova A.S., Pashinkin A.S., Fedorov V.A. (2008). Heat capacity of the n-Bi_2_Te_2.88_Se_0.12_ and p-Bi_0.52_Sb_1.4_8Te_3_ solid solutions. Inorg. Mater..

[20] Kusagaya K., Takashiri M. (2015). Investigation of the effects of compressive and tensile strain on n-type bismuth telluride and p-type antimony telluride nanocrystalline thin films for use in flexible thermoelectric generators. J. Alloys Compd..

[21] Lotgering F.K. (1959). Topotactical Reactions with Ferrimagnetic Oxides Having Hexagonal Crystal Structures—I.

[22] Ionescu R., Jaklovszky J., Nistor N., Chiculita A. (1975). Grain size effects on thermoelectrical properties of sintered solid solutions based on Bi_2_Te_3_. Phys. Status Solidi (A).

[23] Kavei G., Karami M.A. (2008). Formation of anti-site defects and bismuth overstoichiometry in p-type Sb_2−x_BixTe_3_ thermoelectric crystals. Eur. Phys. J. Appl. Phys..

[24] Navrátil J., Starý Z., Plecháček T. (1996). Thermoelectric properties of p-type antimony bismuth telluride alloys prepared by cold pressing. Mater. Res. Bull..

[25] Martin J., Wang L., Chen L., Nolas G.S. (2009). Enhanced Seebeck coefficient through energy-barrier scattering in PbTe nanocomposites. Phys. Rev. B Condens. Matter. Mater. Phys..

[26] Ioannou M., Polymeris G.S., Hatzikraniotis E., Paraskevopoulos K.M., Kyratsi T. (2014). Effect of Bi-doping and Mg-excess on the thermoelectric properties of Mg_2_Si materials. J. Phys. Chem. Solids.

[27] Symeou E., Karyou M., Delimitis A., Constantinou M., Constantinides G., Nicolaou C., Giapintzakis I., Kyratsi T. (2022). Preparation of highly efficient thermoelectric Bi-doped Mg_2_Si_0.55−x_Sn_0.4_Ge_x_ (x = 0 and 0.05) materials with a scalable mechanical alloying method. J. Phys. Chem. Solids.

[28] Jiang J., Chen L., Bai S., Yao Q., Wang Q. (2005). Thermoelectric properties of p-type (Bi_2_Te_3_)_x_(Sb_2_Te_3_)_1−x_ crystals prepared via zone melting. J. Cryst. Growth.

[29] Cahill D.G., Watson S.K., Pohl R.O. (1992). Lower limit to the thermal conductivity of disordered crystals. Phys. Rev. B.

[30] Neophytou N., Vargiamidis V., Foster S., Graziosi P., Oliveira L.d.S., Chakraborty D., Li Z., Thesberg M., Kosina H., Bennett N. (2020). Hierarchically nanostructured thermoelectric materials: Challenges and opportunities for improved power factors. Eur. Phys. J. B.

[31] Qiu B., Ruan X. (2009). Molecular dynamics simulations of lattice thermal conductivity of bismuth telluride using two-body interatomic potentials. Phys. Rev. B Condens. Matter Mater. Phys..

[32] Hellman O., Broido D.A. (2014). Phonon thermal transport in Bi2 Te3 from first principles. Phys. Rev. B Condens. Matter Mater. Phys..

[33] Wang S., Lu X., Negi A., He J., Kim K., Shao H., Jiang P., Liu J., Hao Q. (2022). Revisiting the Reduction of Thermal Conductivity in Nano- to Micro-Grained Bismuth Telluride: The Importance of Grain-Boundary Thermal Resistance. Eng. Sci..

[34] Nan C.W., Birringer R. (1998). Determining the Kapitza resistance and the thermal conductivity of polycrystals: A simple model. Phys. Rev. B.

[35] Nandihalli N., Mori T., Kleinke H. (2018). Effect of addition of SiC and Al_2_O_3_ refractories on Kapitza resistance of antimonide-telluride. AIP Adv..

[36] Tarkhanyan R.H., Niarchos D.G. (2014). Effect of Pore Size on Reduction of the Lattice Thermal Conductivity of Nano to Micro-Scale Porous Materials. J. Electron. Mater..

[37] Tarkhanyan R.H., Niarchos D.G. (2013). Reduction in lattice thermal conductivity of porous materials due to inhomogeneous porosity. Int. J. Therm. Sci..

[38] Wang Z., Alaniz J.E., Jang W., Garay J.E., Dames C. (2011). Thermal conductivity of nanocrystalline silicon: Importance of grain size and frequency-dependent mean free paths. Nano Lett..

[39] Chakraborty D., de Sousa Oliveira L., Neophytou N. (2019). Enhanced Phonon Boundary Scattering at High Temperatures in Hierarchically Disordered Nanostructures. J. Electron. Mater..

[40] Kanno T., Tamaki H., Sato H.K., Kang S.D., Ohno S., Imasato K., Kuo J.J., Snyder G.J., Miyazaki Y. (2018). Enhancement of average thermoelectric figure of merit by increasing the grain-size of Mg_3.2_Sb_1.5_Bi_0.49_Te_0.01_. Appl. Phys. Lett..

[41] Wei T.-R., Tan G., Zhang X., Wu C.-F., Li J.-F., Dravid V.P., Snyder G.J., Kanatzidis M.G. (2016). Distinct Impact of Alkali-Ion Doping on Electrical Transport Properties of Thermoelectric p-Type Polycrystalline SnSe. J. Am. Chem. Soc..

[42] Qiu Q., Liu Y., Xia K., Fang T., Yu J., Zhao X., Zhu T. (2019). Grain Boundary Scattering of Charge Transport in n-Type (Hf,Zr)CoSb Half-Heusler Thermoelectric Materials. Adv. Energy Mater..

[43] Son J.H., Oh M.W., Kim B.S., Park S.D. (2018). Optimization of thermoelectric properties of n-type Bi_2_(Te,Se)_3_ with optimizing ball milling time. Rare Met..

[44] Kuo J.J., Wood M., Slade T.J., Kanatzidis M.G., Snyder G.J. (2020). Systematic over-estimation of lattice thermal conductivity in materials with electrically-resistive grain boundaries. Energy Environ. Sci..

